# Enhanced Power Output of a Triboelectric Nanogenerator Composed of Electrospun Nanofiber Mats Doped with Graphene Oxide

**DOI:** 10.1038/srep13942

**Published:** 2015-09-21

**Authors:** Tao Huang, Mingxia Lu, Hao Yu, Qinghong Zhang, Hongzhi Wang, Meifang Zhu

**Affiliations:** 1 State Key Laboratory for Modification of Chemical Fibers & Polymer Materials, College of Materials Science & Engineering, Donghua University, Shanghai 201620, P. R. China; 2 Shanghai Key Laboratory of Functional Hybrid Materials, College of Materials Science & Engineering, Donghua University, Shanghai 201620, P. R. China; 3 Engineering Research Center of Advanced Glasses Manufacturing Technology, College of Materials Science & Engineering, Donghua University, Shanghai 201620, P. R. China

## Abstract

We developed a book-shaped triboelectric nanogenerator (TENG) that consists of electrospun polyvinylidene fluoride (PVDF) and poly(3-hydroxybutyrate-co-3-hydroxyvalerate) (PHBV) nanofibers to effectively harvest mechanical energy. The dispersed graphene oxide in the PVDF nanofibers acts as charge trapping sites, which increased the interface for charge storage as well as the output performance of the TENG. The book-shaped TENG was used as a direct power source to drive small electronics such as LED bulbs. This study proved that it is possible to improve the performance of TENGs using composite materials.

Scavenging energy from various ambient mechanical vibrations and motions is considered feasible as a promising green technology to realize self-powered mobile and wearable electronic devices^1^,^2^,^3^,^4^. In 2012, Wang’s group demonstrated a novel energy harvester named the triboelectric nanogenerator (TENG) that converts environmental mechanical energy into electric energy. Since then, various TENGs based on the triboelectric effect and electrostatic induction have been developed as an energy source for applications including an electrochromic device^5^, water splitting^6^, portable electronics^7^,^8^,^9^,^10^,^11^,^12^,^13^,^14^,^15^, and self-powered sensors^16^,^17^,^18^,^19^,^20^,^21^,^22^,^23^ without reliance on traditional power supplies. Subsequently, various important factors for improving the output power of the TENG itself have been studied, including (1) functional materials^7^,^24^, (2) surface microstructure design^25^, and (3) optimization of device structure^15^,^16^,^21^,^26^,^27^,^28^,^29^,^30^,^31^,^32^. Point (1) has been realized by selecting suitable functional materials, *e.g*., polydimethylsiloxane, polytetrafluoroethylene, and polyethylene terephthalate (PET), according to the triboelectric series. Previous studies indicate that to achieve high performance, researchers should select materials that are far away from each other in the series to obtain a large difference in electronegativity. Instead of using relatively flat polymer sheets, micro/nano patterns have been fabricated on the polymer surfaces to increase the interface roughness of the friction surfaces, which generates more triboelectric charges at the surface and allows them to be separated more easily because the contact area of the surface is decreased. Another consideration is that the effective periodic switching between separation and intimate contact of the two charged surfaces is important to determine the electrostatic potential across the two electrodes, which is the driving force for the free electrons. Thus, various structures like arch- and sandwich-shaped TENGs and inclusion of a spacer have been used to achieve effective charge separation. However, on the one hand, although only TENGs composed of single materials have been reported, we believe that the contact materials can be composites, such as nanoparticles embedded in a polymer matrix. This would not only change the surface electrification and permittivity of the materials so that they can be more effective at electrostatic induction to improve the performance of the TENG, but also expand the choice of the triboelectric material from single ones to composites, which would provide further ways to enhance the performance of the TENG according to point (1). On the other hand, all the reported approaches to prepare TENGs require somewhat complicated, expensive fabrication methods, like photolithography or reactive ion etching, or additional processes, *e.g*., electrode preparation, and have limited scalability for mass production. To use TENGs in diverse applications, a simple, cost-effective and readily scalable fabrication process for efficient TENGs needs to be developed.

Electrospinning is a versatile, simple method to process polymer solutions or melts into ultrathin fibers with a number of unique features and properties^33^,^34^. This cost-effective method also provides the capacity to lace together natural polymers, polymer alloys, and polymers loaded with chromophores, inorganic nanoparticles, or active agents, as well as metals and ceramics to produce multifunctional nonwoven nanocomposite membranes with advanced characteristics not observed for single materials^35^,^36^,^37^. In consequence, electrospinning has proved to be a suitable method to fabricate triboelectric materials^38^.

In this work, we design a book-shaped TENG that is fabricated from polyvinylidene fluoride (PVDF) and poly(3-hydroxybutyrate-co-3-hydroxyvalerate) (PHBV) nanofibers *via* electrospinning. The nanofiber structure can efficiently generate triboelectric charges on its surface without any additional complex or expensive processes to tailor microstructure. In addition, the aluminum foil used in electrospinning also acts as the electrode of the TENG, which removed the need for an electrode preparation process. Graphene oxide (GO) is embedded in the PVDF matrix to modify the surface electrification of the triboelectric materials and enhance the output performance of the TENG.

## Results and Discussion

**Figure 1:**
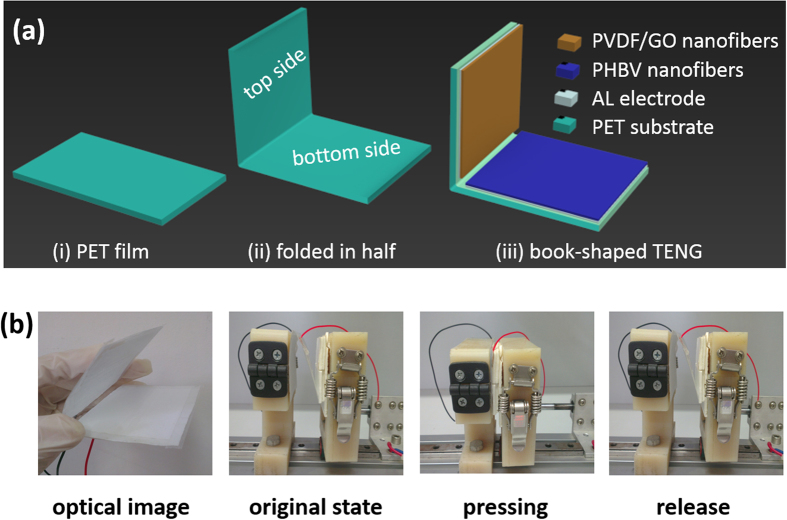
Structure and fabrication process of a book-shaped TENG. (**a)** Schematic diagram of a TENG on a folded PET substrate. (**b)** Photographs of a TENG in its initial, compressed and released states.

This book-shaped TENG is based on the contact electrification between PVDF as the electronegative material and PHBV as the electropositive material. PVDF and PHBV were carefully chosen according to the triboelectric series (supplementary information, Fig. S2)^39^ to possess a very large difference in their ability to attract and retain electrons, and more importantly, their ease of fabrication into nanofibers. The book-shaped structure of the TENG helps to realize the action of effective charge separation and contact using the natural elasticity of the bent PET substrate (Fig. 1a). The working mode of the book-shaped TENG is carried out by applying a cycled compressive force to the whole area of the device during the periodic pressing and release motion of the pressing stage (Fig. 1b) so that the top and bottom of the PET substrate coated with PVDF and PHBV nanofibers are periodically pressed into close contact with each other. Once released, the two sides separate automatically because of the stored elastic energy of the PET substrate.

**Figure 2:**
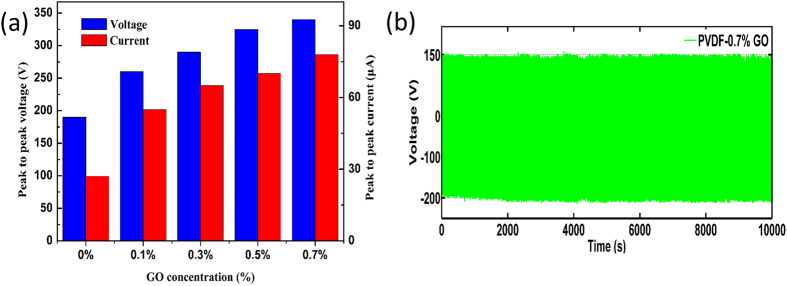
Performance of TENGs with different GO contents. (**a)** Output voltage and current of book-shaped TENGs containing PVDF nanofibers with different weight ratios of GO. The current was measured under an external load of 100 kΩ. (**b)** Durability of a book-shaped TENG containing PVDF nanofibers with 0.7% GO over 18000 cycles.

The output voltage and current of the fabricated book-shaped TENG under the external force of the pressing stage operating at a frequency of 1.8 Hz were investigated. As displayed in Fig. 2a, under continual pressing and release cycles, the TENG composed of PHBV and PVDF nanofibers without GO repeatedly generated a peak-to-peak voltage of 190 V and peak-to-peak current of 27 μA. Interestingly, the voltage and current increased sharply when a small amount of GO was included in the PVDF nanofibers. The generated peak-to-peak voltage and current reached 260 V and 55 μA, respectively, for the sample containing 0.1% GO in PVDF. The performance of the TENG was further improved by increasing the content of GO in the PVDF nanofibers. The maximum output peak-to-peak voltage and current reached 340 V and 78 μA, respectively, when the GO concentration was 0.7%, which are increases of 78.9% and 188.9%, respectively, compared with those of the TENG containing bare PVDF nanofibers (Fig. S3 and S4). The durability of the book-shaped TENG containing PVDF nanofibers doped with 0.7% GO was assessed, as presented in Fig. 2b. The TENG showed little change in performance after 18000 cycles, indicating that the nanofiber structures were not damaged after thousands of contact-and-release motions, unlike TENGs fabricated by traditional complex or expensive methods such as lithography, etching, and microimprinting^26^.

**Figure 3:**
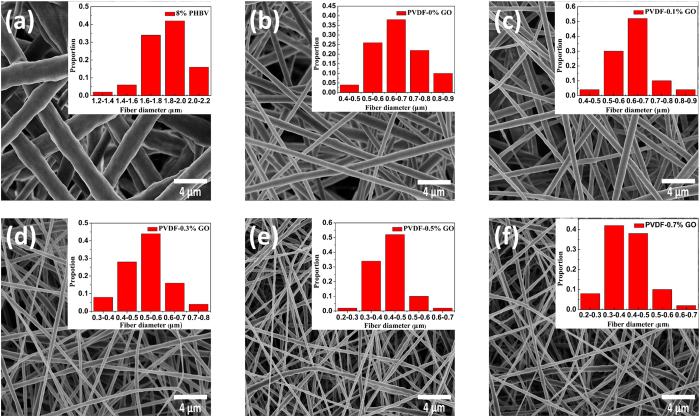
Structure of the nanofibers used in the book-like TENGs. Morphology and size distributions (inset) of 8% PHBV and 10% PVDF nanofibers with different weight ratios of GO: (**a**) 8% PHBV, (**b**) PVDF with 0% GO, (**c**) PVDF with 0.1% GO, (**d**) PVDF with 0.3% GO, (**e**) PVDF with 0.5% GO, and (**f**) PVDF with 0.7% GO.

**Figure 4:**
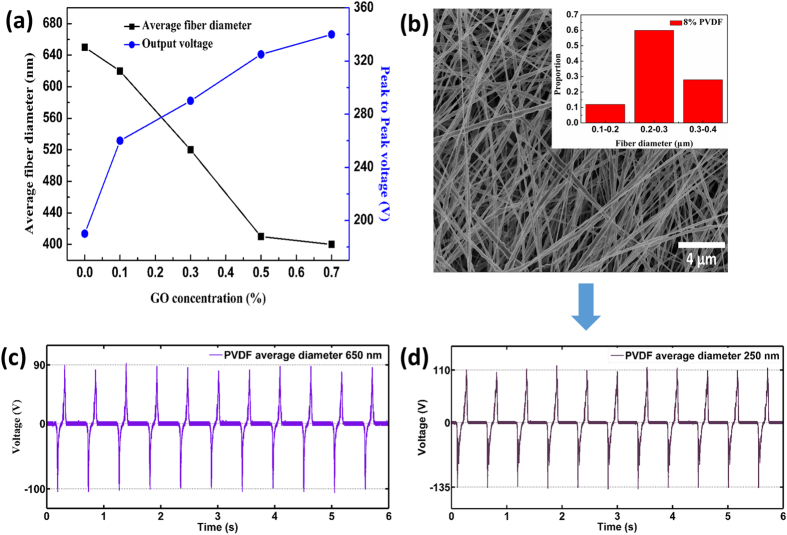
Influence of GO concentration and fiber diameter on output voltage. (**a**) Dependence of average fiber diameter and output peak-to-peak voltage on GO concentration. (**b**) FE-SEM image and size distribution (inset) of PVDF nanofibers fabricated using a spinning solution with a PVDF concentration of 8%. Peak-to-peak voltages of bare PVDF nanofibers with an average fiber diameter of (**c**) 650 nm and (**d**) 250 nm.

The surface structure of triboelectric materials strongly affects the output voltage of the corresponding TENG^11^,^15^. Thus, to find out the reason for the enhanced output voltage of the book-like TENG, the surface morphology of the triboelectric materials were studied (Fig. 3). The morphology of the PHBV nanofibers was kept the same in our devices to simplify the analysis, as presented in Fig. 3a. Interestingly, as shown in Fig. 3b–f, the average fiber diameter of the PVDF/GO nanofibers decreased from 650 nm for pure PVDF to 400 nm for PVDF containing 0.7% GO (Fig. 4a). Therefore, the enhanced output voltage of the TENG with increased GO content was possibly caused by the diameter of the PVDF/GO fibers decreasing as their content of GO increased. Thus, a control experiment was conducted to verify this conjecture. A TENG containing bare PVDF nanofibers with an average diameter of 250 nm was prepared to examine the potential effect of fiber diameter (Fig. 4b). If the performance of the TENGs had nothing to do with GO, the generated peak-to-peak voltage of this device should have been over 340 V according to the curve in Fig. 4a. However, the actual output peak-to-peak voltage of the TENG containing 250-nm PVDF nanofibers was 245 V. That is, the peak-to-peak voltage generated by bare PVDF nanofibers only increased by 28.9% (from 190 to 245 V) when the diameter was decreased by 61.5% (from 650 to 250 nm) (Fig. 4c,d). This result indicates that the decrease in diameter of the bare PVDF nanofibers did improve the output voltage of the TENG to some extent, but the decrease in diameter was not the main origin of the improved performance. To further identify the contribution of GO to the performance of the TENGs, a series of PVDF/GO cast films without any nanostructures on their surfaces were fabricated (Fig. S5) and the output voltages of the TENGs containing these film were measured (Fig. S6). The increase of voltage of these devices was in accordance with that of the PVDF/GO nanofiber-based TENGs. This indicated that the voltage increase was not caused by GO changing the morphology of the samples. Therefore, it was the GO in the PVDF nanofibers or cast films that played an important role in the performance enhancement of the TENGs.

**Figure 5:**
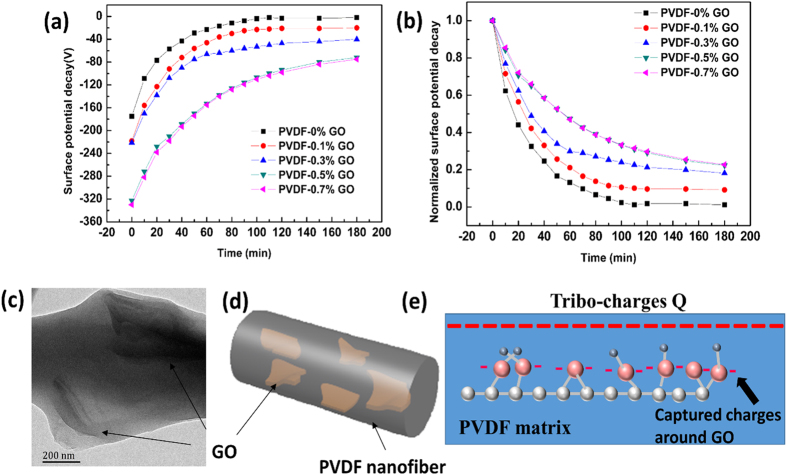
Origin of the effect of GO on TENG performance. (**a**) Surface potential decay and (**b**) the normalized surface potential decay of PVDF/GO nanofibers with different concentrations of GO. (**c**) TEM images of PVDF nanofibers containing 0.7% GO. Schematic diagrams of PVDF/GO nanofibers showing the (**d**) distribution of GO on a nanofiber, and (**e**) stored charge on the surface of a GO sheet.

The mechanisms of TENGs are based on triboelectrification and electrostatic induction, as shown in Fig. S7. Electrons were induced to flow back and forth through the external circuit under the driving force of the change in triboelectric potential between the PHBV and PVDF/GO nanofibers, which produces the observed voltage–current behavior (Fig. S3c) until the TENG reaches electrical equilibrium (Fig. S7d and b). Niu *et al.*^40^ reported that the output voltage and current of a TENG are positively correlated with surface charge densities. Therefore, we can deduce that for a specific TENG, the greater the charge distributed on the surface of the triboelectric materials, the higher the surface potential will be, which leads to a larger driving force for the transfer of electrons as well as the higher observed voltage and current. The surface potential of the PVDF/GO nanofibers that resulted from the triboelectric effect between PVDF/GO and PHBV nanofibers was characterized to confirm this deduction. Fig. 5(a,b) reveal that the surface potential of the PVDF/GO nanofibers becomes larger as the concentration of GO increases, which is in accordance with the output voltage. In addition, the decay of the surface potential is slowed down when the PVDF nanofibers contain more GO. This phenomenon indicates that the improved output voltage of the TENG is caused by the increased surface charge of PVDF/GO nanofibers that resulted from the dispersed GO in the PVDF nanofibers. The improved surface potential (charge) of PVDF/GO compared with that of PVDF alone can be explained as follows. GO is one of the most important derivatives of graphene and possesses a layered structure composed of a carbon network of hexagonal rings and oxygen functional groups on the basal planes and edges^41^,^42^. The dispersed GO in the PVDF nanofibers acts as charge trapping sites, which increased the interface for charge storage. Electrons attracted from PHBV nanofibers through the friction process were stored either in the discrete, quantized levels of these nanosized graphene particles, or trapped in the amorphous GO dielectric^43^,^44^, as shown in Fig. 5c–e. This both increased the surface charge on the PVDF/GO nanofibers, and slowed the dissipation of surface charge.

The charge storage property of GO was another reason for the decrease of diameter of the PVDF/GO fibers. The factors that influence the diameter of electrospun nanofibers have been studied extensively^34^,^45^,^46^,^47^, and include: (1) the intrinsic properties of the polymer and solvent such as the type and molecular weight of the polymer, and the polarity and boiling point of the solvent; (2) solution parameters such as its concentration, viscosity, and electrical conductivity; (3) operating conditions including the strength of the applied electric field, feed rate, and the collection distance; (4) environmental conditions like temperature and relative humidity. However, in our case, the only factors influencing the fibers were the solution parameters; all other parameters were fixed in the spinning process. Thus, we characterized the viscosity and electrical conductivity of the PVDF spinning solutions containing different weight ratios of GO. However, the changes of electrical conductivity (Table S1) and viscosity (Fig. S8) of the PVDF/GO solutions with different contents were so small that these factors cannot markedly affect the fiber diameter. Instead, as discussed above, the dispersed GO sheet could be easily charged when a high voltage was applied to the spinning solution, leading to increases of electrostatic repulsion and Coulombic force on the Taylor cone, which caused the fiber diameters to decrease.

**Figure 6:**
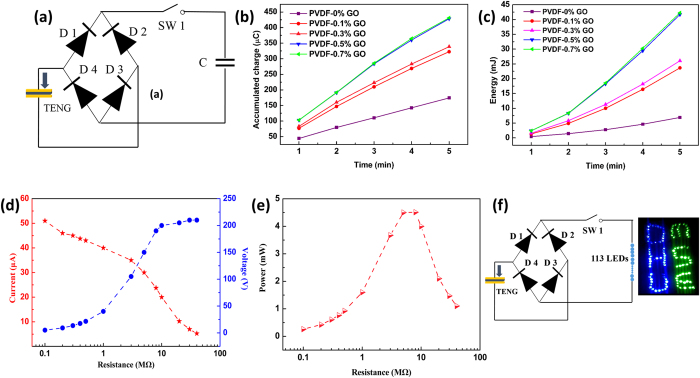
Performance and application of a book-like TENG. (**a**) The circuit used to store energy generated from a book-shaped TENG. (**b**) Accumulated charge and (**c**) stored energy in a 2.2-μF capacitor over 5 minutes (540 cycles). Dependence of (**d**) output voltage and current, and (**e**) instantaneous power on load resistance. (**f**) Schematic diagram of a prototype energy-harvesting circuit and TENG as a direct power source to drive 113 commercial LEDs.

For self-powered systems, the energy generated by a TENG needs to be managed. Fig. 6a shows a full-wave rectifier bridge and 2.2-μF capacitor to store the energy generated by a TENG. Fig. 6(b,c) depicts the accumulated charges and energy across the capacitor over 5 min when powered by different TENGs. As the GO content of the PVDF nanofibers increased, so did the charging power of the TENG, which is in accordance with the output voltage. The charge and energy generated by the bare PVDF nanofibers after 5 min were 174.3 μC and 6.9 mJ, respectively, giving an average charging current of 0.581 μA and average power of 0.023 mW, respectively. In contrast, the charge and energy of PVDF nanofibers containing 0.7% GO were 431.3 μC and 42.3 mJ, respectively. These values gave an average charging current of 1.438 μA and average power of 0.141 mW, which are increases of 147.5% and 513.0%, respectively.

In practical use, the output power for a load depends on the resistance of the load itself. Therefore, we characterized the variations in output voltage and current of a working TENG composed of PVDF containing 0.7% GO under different external loads from 0.1–40 MΩ, as shown in Fig. 6d. As load resistance was increased, the voltage rose and was saturated at about 200 V, which is consistent with the output voltage of the device, while the current decreased because of the ohmic loss. As displayed in Fig. 6e, the instantaneous power on the load reached a maximum value of 4.5 mW at a load resistance of 8 MΩ, corresponding to a power density of 2.3 W/m^2^.

To demonstrate the feasibility of the book-shaped TENG as a direct power source for electronics, a total of 113 commercial LEDs connected in series to form a character sequence of *DHU-MSE* were used as an external load (Supplementary Video 1). As illustrated in Fig. 6f, a bridge rectifier was used to convert the AC output signals into DC signals to ensure that the LEDs could be lighted by the TENG in both the pressing or release state. The photograph of the flashing LEDs was captured while the TENG was being pressed at a frequency of 1.8 Hz by the pressing stage.

In summary, a simple book-shaped TENG composed of PVDF and PHBV nanofibers was developed to harvest mechanical energy. The TENG was fabricated by electrospinning using a facile and cost-effective process. GO sheets were introduced to increase the charge storage ability of the PVDF nanofibers and enhance the output performance of the TENG. The maximum output peak-to-peak voltage and current were 340 V and 78 μA, respectively, and the average charging current and power for charging a 2.2-μF capacitor were 1.438 μA and 0.141 mW, respectively, which are 78.9%, 188.9%, 147.5% and 513.0%, respectively, higher than those of a TENG with neat PVDF nanofibers. The energy generated could either be stored or directly used to drive small electronics and the performance of the TENG was quite stable over 18000 cycles. Importantly, this study demonstrated groundbreaking progress in the enhancement of the output power of TENGs by using inorganic/organic hybrid materials and a simple electrospinning method, which may open new avenues of research for triboelectric materials.

## Methods

### Materials

PVDF (FR904, Mw = 6 × 10^5^) powder was purchased from Shanghai 3F New Material Co., Ltd. PHBV (Y1000P, Mw = 2.67 × 10^5^, HV = 1.10 mol%) was purchased from Zhengjiang Tianan Biological Material Co. Ltd. GO was prepared by the conventional Hummers method^48^. AR grade N,N-dimethylformamide (DMF), trichloromethane and acetone were obtained from the Shanghai Chemical Reagent Plant.

### Electrospinning

Different amounts of GO (1–7 mg) were ultrasonically dispersed in DMF/acetone (3/2 w/w, 9 g) for 2 h. PVDF was dissolved in the resulting suspensions to give a polymer/solvent weight ratio of 1:9 (10%, w/w). Each mixture was stirred for 12 h to form a homogenous solution. PHBV was dissolved in trichloromethane at 60 °C to prepare PHBV solution (8%, w/w). In a custom-made electrospinning set-up (Fig. S1), a polymer solution was charged into a syringe that was subjected to a DC voltage if up to 18 kV by DC power supply (JG50-1, Shanghai Shenfa Detection Instrument, China). The syringe was placed in a microsyringe pump (KDS101, KD Scientific, USA) and the spinning solution was delivered to the blunt needle tip at a flow rate of 1 mL h^−1^ at a fixed collection distance of 15 cm between the tip of the syringe and roller collector. The relative humidity was controlled below 30%.

### Fabrication of book-shaped TENGs

A rectangular PET film (10 cm × 6 cm × 100 μm) was folded in half in the length direction (Fig. 1a(I,ii)). Pieces of PVDF or PHBV nanofiber mat (4 × 5 cm) were pasted on the top or bottom of the PET film (Fig. 1a(iii)). The aluminum foil that was used to collect the nanofibers during electrospinning acted as the electrode for the TENG.

### Characterization

The morphology of the electrospun fiber structure was examined by field-emission scanning electron microscopy (FESEM; S-4800, Hitachi, Japan). A thin platinum layer was sputtered on the sample surface before the examination. PVDF/GO nanofibers were observed by transmission electron microscopy (TEM; JEM-2100, JEOL, Japan) after being directly electrospun onto a copper grid coated with a holey carbon film. The electrical conductivities of the solutions were measured using a conductivity meter (EL-30, Mettler Toledo) equipped with a sensor probe (Lnllab741) at room temperature. The shear viscosity of the PVDF/GO solutions was detected using a rheometer (R/S Plus, Brookfield) under shear rates from 0 to 300 s^−1^ at 25 °C. An oscillometer (LeCroy, Wavesurfer 104MXs-B) was used to collect the electrical signals generated by the book-shaped TENGs under the force of the bending stage at a frequency of 1.8 Hz. A Keithley 2400 meter was used to measure the voltage across the capacitors. The surface potential of the PVDF/GO nanofibers was detected by an electrostatic voltmeter (Trek, 541A).

## Additional Information

**How to cite this article**: Huang, T. *et al.* Enhanced Power Output of a Triboelectric Nanogenerator Composed of Electrospun Nanofiber Mats Doped with Graphene Oxide. *Sci. Rep.* **5**, 13942; doi: 10.1038/srep13942 (2015).

## Supplementary Material

Supplementary Information
