# Supplementary material for: Enhanced Power Output of a Triboelectric Nanogenerator Composed of Electrospun Nanofiber Mats Doped with Graphene Oxide
Source: Sci Rep. 2015 Sep 21;5:13942. doi: 10.1038/srep13942 (PMC4585669; doi:10.1038/srep13942)
Supplement: Supplementary Information [file srep13942-s1.doc]

Supplementary Information

Enhanced Power Output of a Triboelectric Nanogenerator Composed of Electrospun Nanofiber Mats Doped with Graphene Oxide

Tao Huang^1^, Mingxia Lu^1^, Hao Yu^*1^, Qinghong Zhang^2^, Hongzhi Wang^3^, Meifang Zhu^1^

^1^State Key Laboratory for Modification of Chemical Fibers & Polymer Materials, College of Materials Science & Engineering, Donghua University, Shanghai 201620, P. R. China.

^2^Shanghai Key Laboratory of Functional Hybrid Materials, College of Materials Science & Engineering, Donghua University, Shanghai 201620, P. R. China.

^3^Engineering Research Center of Advanced Glasses Manufacturing Technology, College of Materials Science & Engineering, Donghua University, Shanghai 201620, P. R. China.

^*^Correspondence and requests for materials should be addressed to H. Yu ([yuhao@dhu.edu.cn](mailto:yuhao@dhu.edu.cn))


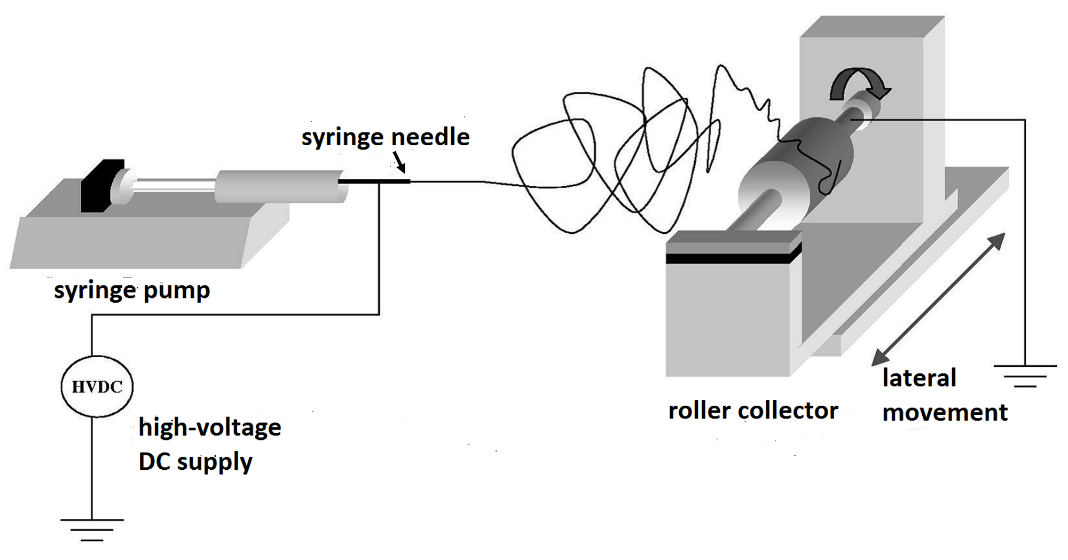


**Supplementary Figure S1.** **Schematic of the homemade electrospinning apparatus**. The nanofibers were collected on the surface of a roller collector covered with aluminum foils.


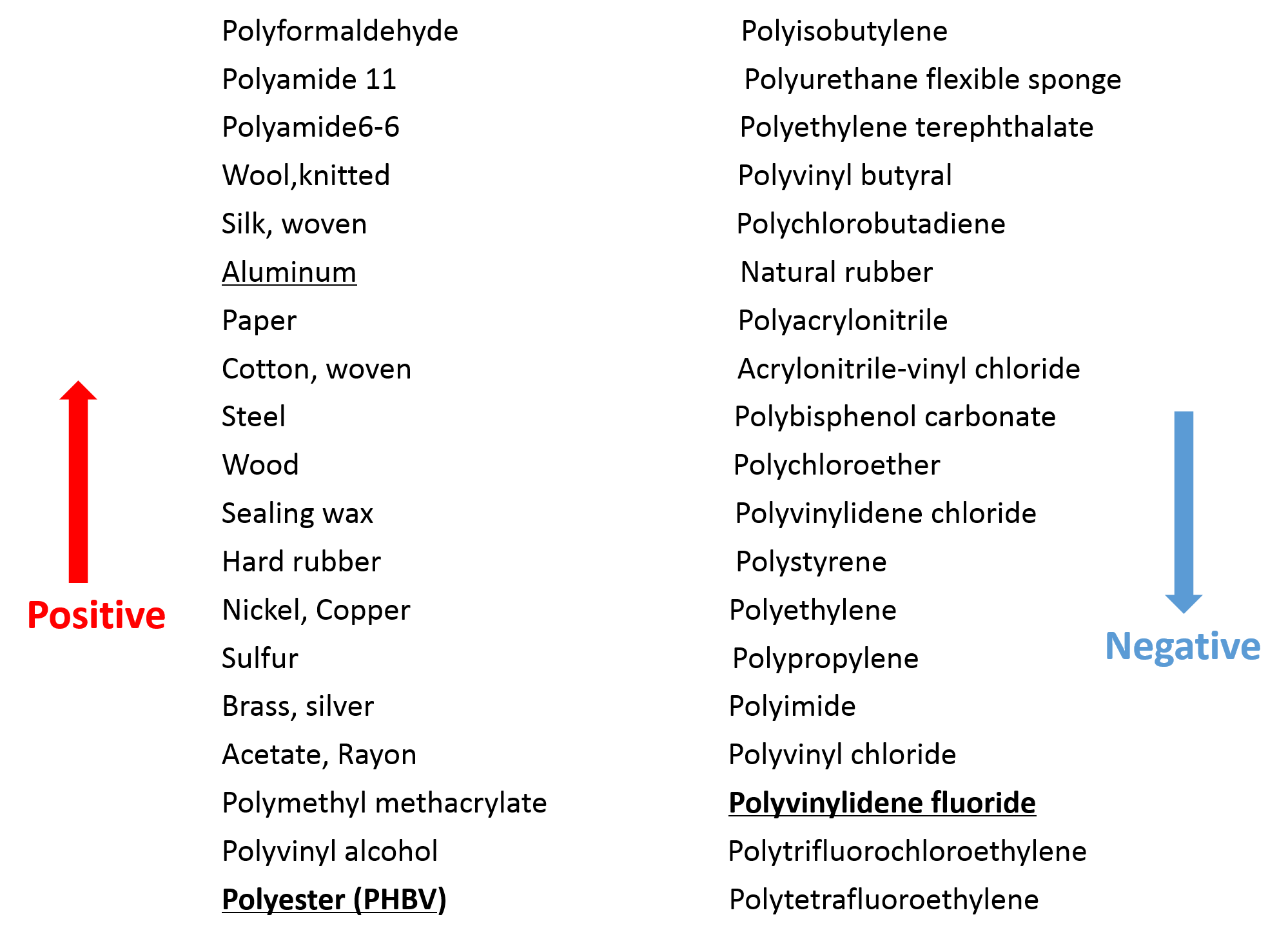


**Supplementary Figure S2.** **The triboelectric series:** a list that ranks various materials according to their tendency to gain (negative) or lose electrons (positive) in contact charging and frictional charging process. Polyvinylidene fluoride (PVDF) is one of the most negative ones, and polyester on behalf of PHBV are tend to lose electrons.


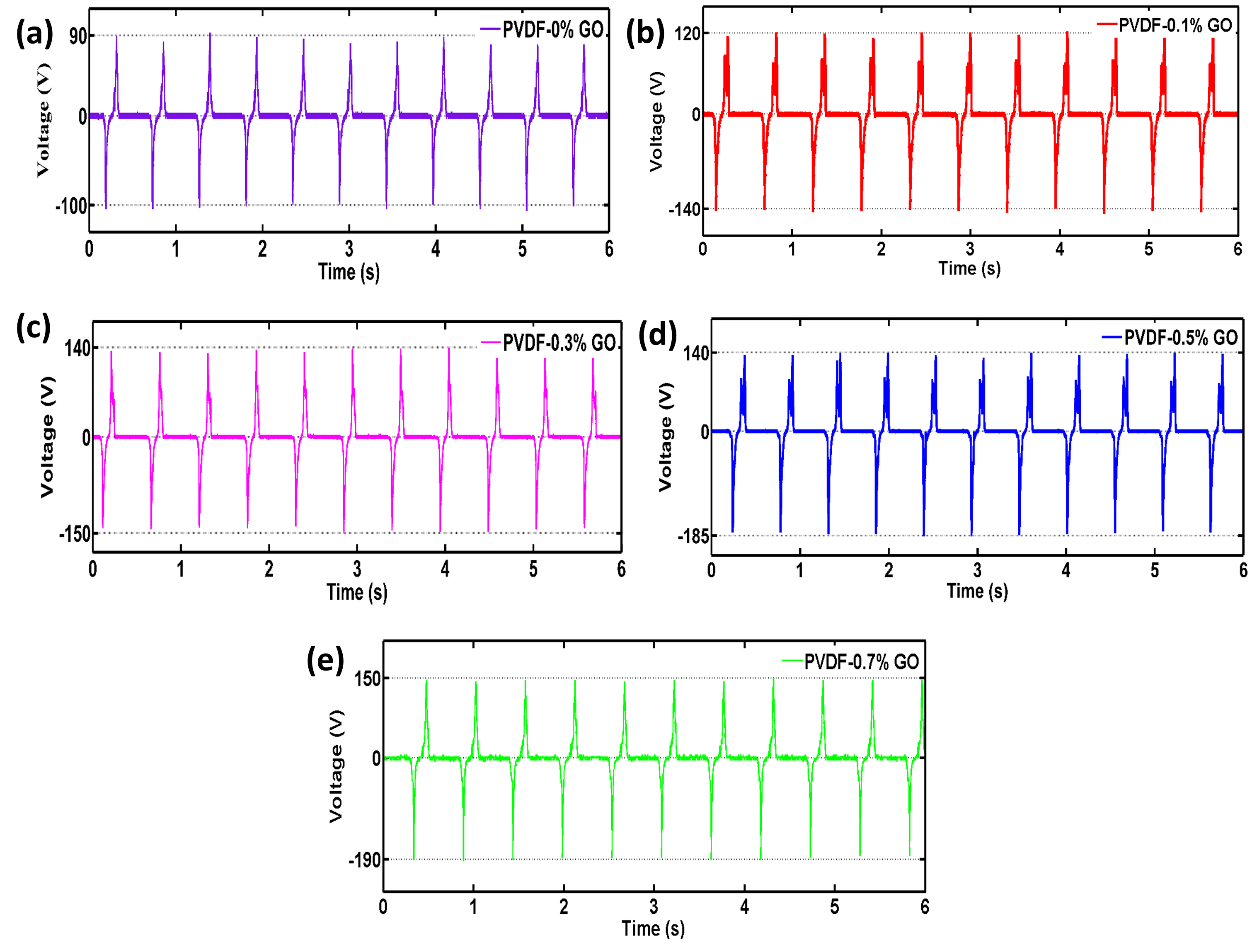


**Supplementary Figure S3. Output voltage of a book-shaped TENG.** The output voltage of book-shaped TENG fabricated by PVDF nanofibers with different weight ratios of GO dosages. **a** PVDF with 0% GO, **c** PVDF with 0.1% GO, **d** PVDF with 0.3% GO, **e** PVDF with 0.5% GO, and **f** PVDF with 0.7% GO.


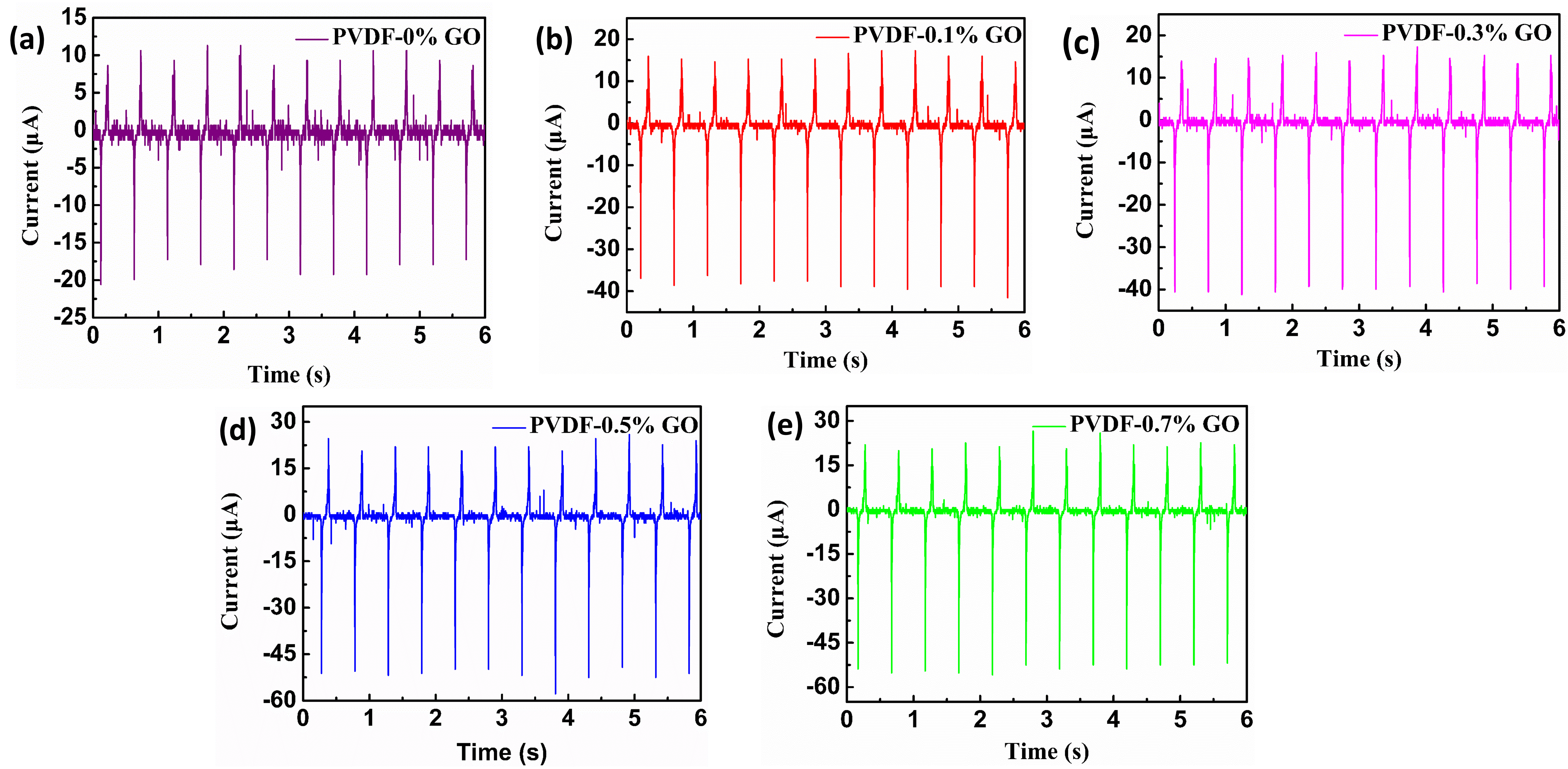


**Supplementary Figure S4. Output current of a book-shaped TENG.** The output current of book-shaped TENG fabricated by PVDF nanofibers with different weight ratios of GO dosages under an external load of 100kΩ. **a** PVDF with 0% GO, **c** PVDF with 0.1% GO, **d** PVDF with 0.3% GO, **e** PVDF with 0.5% GO, and **f** PVDF with 0.7% GO.


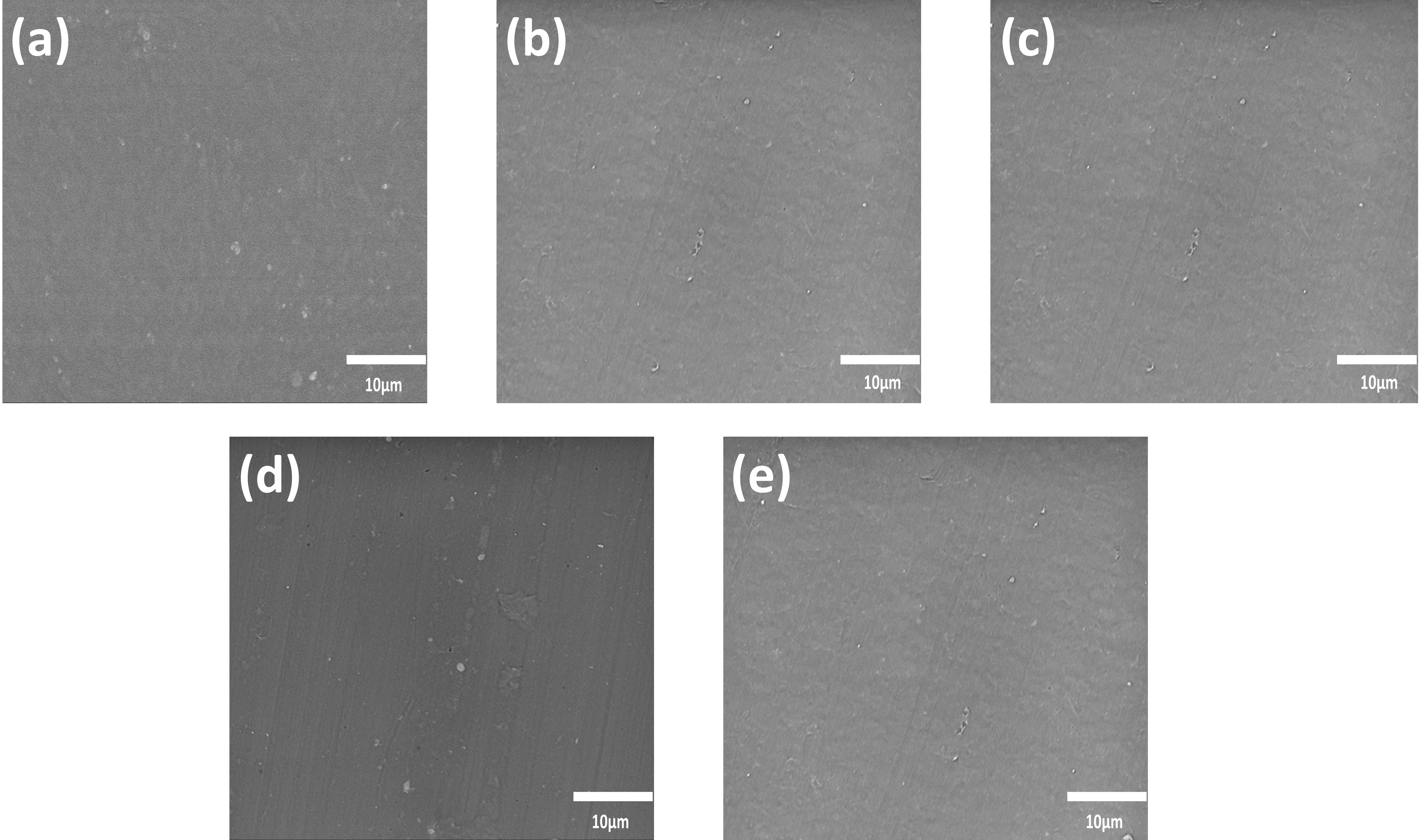


**Supplementary Figure S5. The surface morphology of PVDF cast films with different weight ratios of GO dosages confirming their uniform structures**: **a** PVDF cast film with 0% GO; **b** PVDF cast film with 0.1% GO; **c** PVDF cast film with 0.3% GO; **d** PVDF cast film with 0.5% GO; **e** PVDF cast film with 0.7% GO.


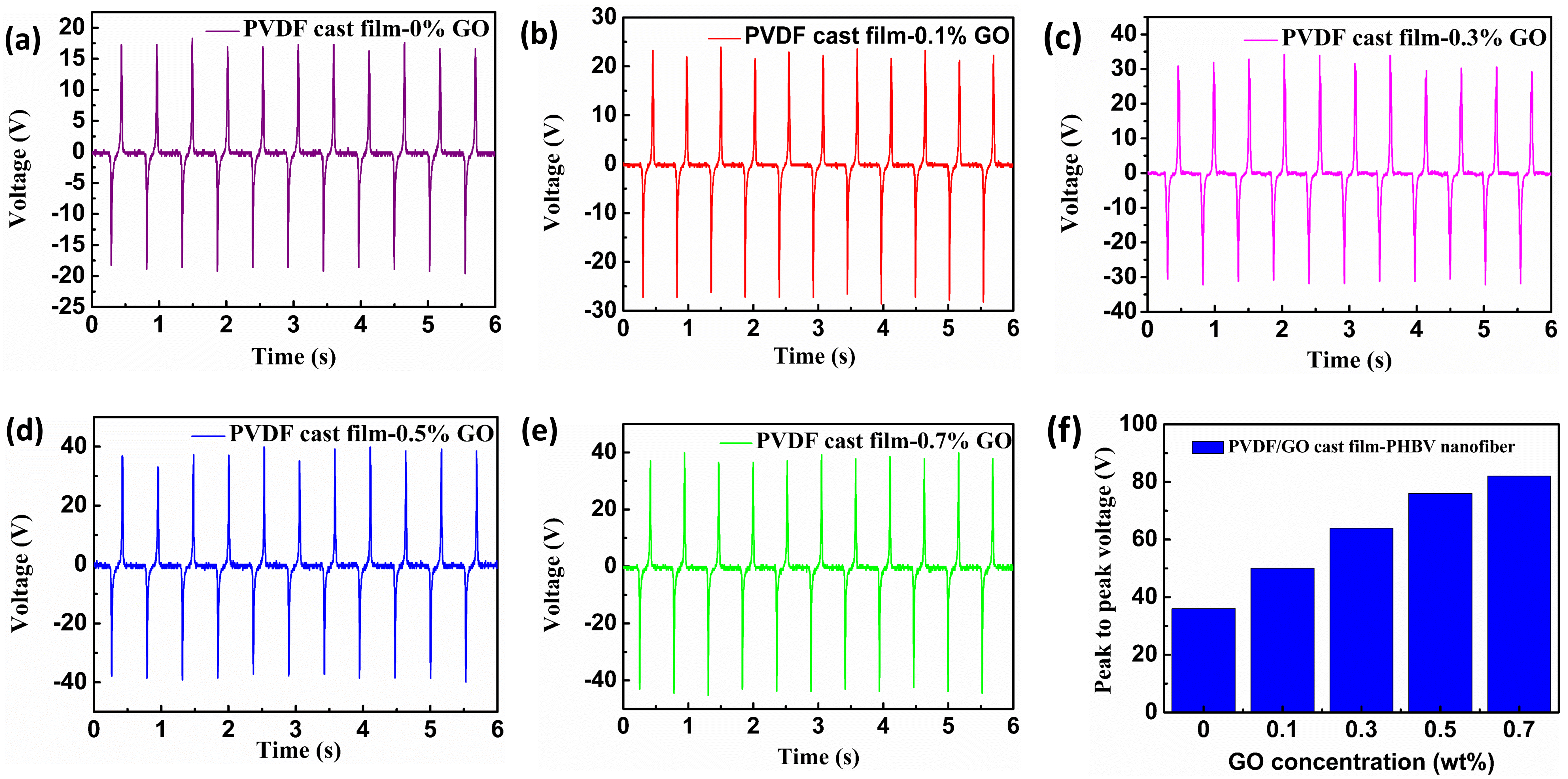


**Supplementary Figure S6. Output voltage of a film based book-shaped TENG.** The output voltage of book-shaped TENG fabricated by PVDF cast films with different weight ratios of GO dosages **a** PVDF cast film with 0% GO; **b** PVDF cast film with 0.1% GO; **c** PVDF cast film with 0.3% GO; **d** PVDF cast film with 0.5% GO; **e** PVDF cast film with 0.7% GO; **f** The bar chart directly showing the voltage changes with the GO concentrations.


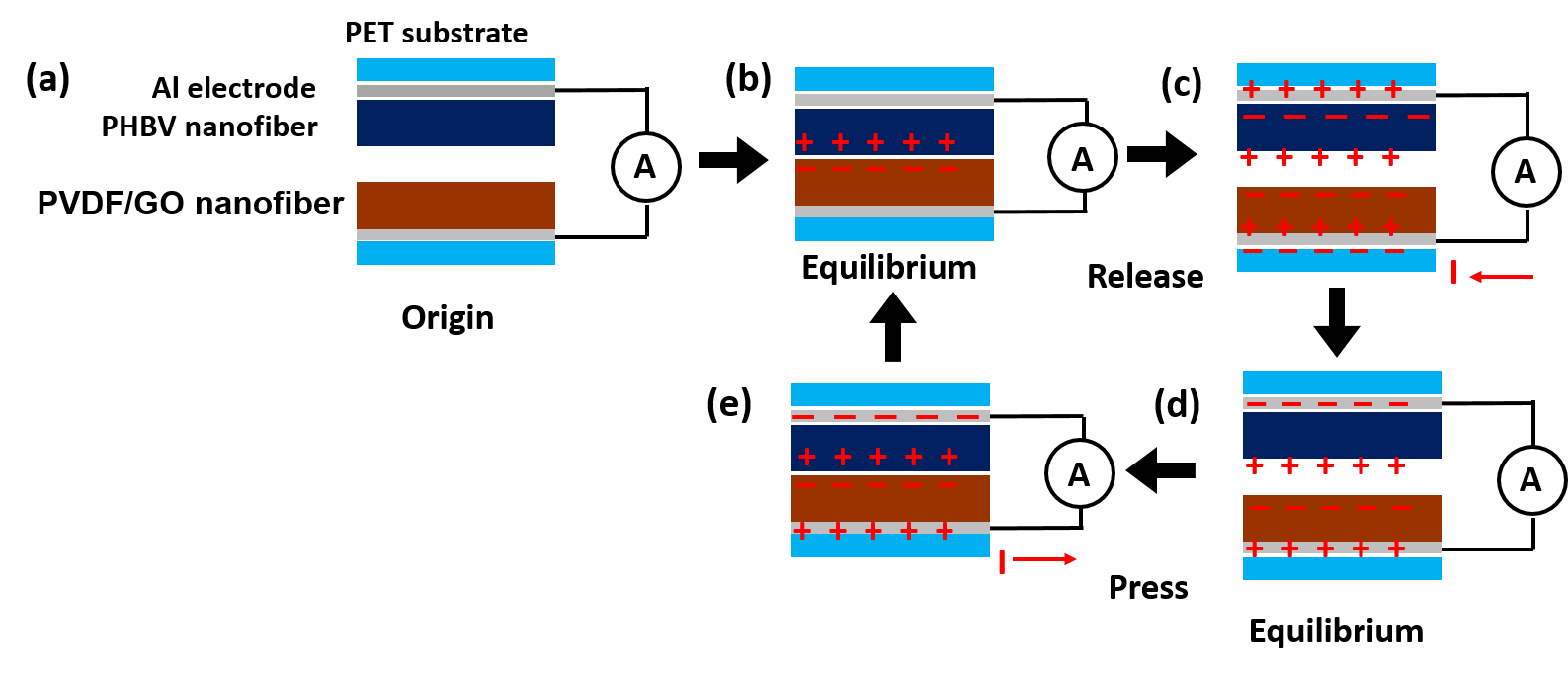


**Supplementary Figure S7.** **Schematics of the power generation mechanism of the TENG device.** **a** The initial state of the device; **b** With a pressing force and the contact of PVDF and PHBV, electrostatic triboelectric charges with opposite signs are generated and distributed on the two surfaces of the nanofibers; **c** Once the force is released, the opposite triboelectric charges become separated with an air gap and a dipole moment forms, and the induced electric potential difference will drive the electrons flow across the external load; **d** The electric potential between the planar electrodes reaches equilibrium and electrons accumulate on the one side of the electrodes, leaving the other side positively charged; **e** When another pressing process starts, the dipole moment disappears or reduced and the accumulated electrons will be driven to flow in the opposite direction. Therefore, an alternating electrons flow in the external load will be observed with cycled pressing and releasing process.

**
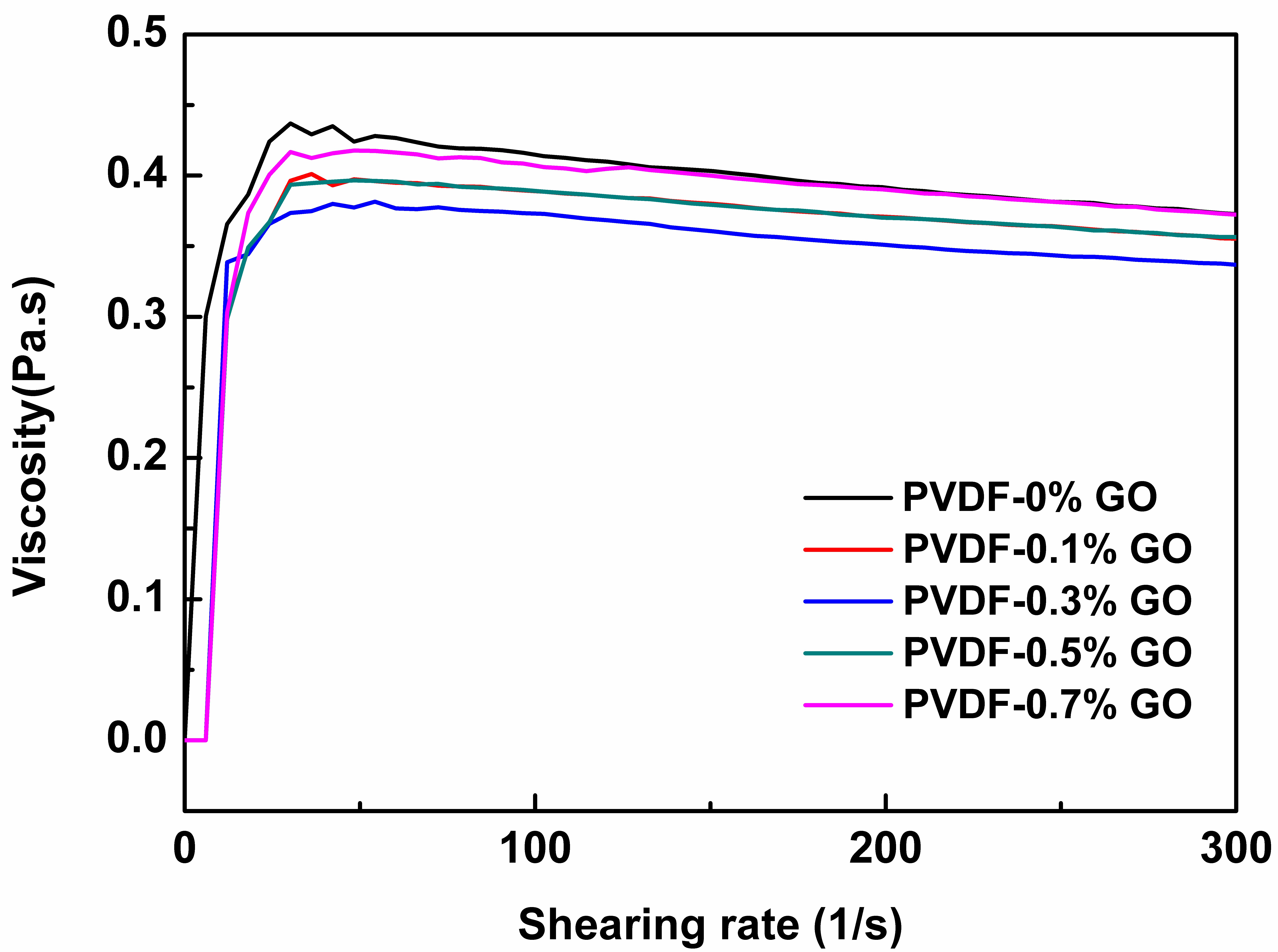
**

**Supplementary Figure S8. Viscosities as a function of shearing rate for PVDF/GO solutions with various GO dosages**

**Table S1.** **The conductivities PVDF/GO solutions with the different weight ratios of GO loadings**

| Concentration (%) | 0 | 0.1 | 0.3 | 0.5 | 0.7 |
| --- | --- | --- | --- | --- | --- |
| Conductivity (μs/cm) | 14.82 | 14.89 | 20.90 | 22.90 | 22.50 |

**Supplementary S9.** **Video 1 Showing the book-shaped TENG driven 113 commercial LEDs by the pressing stage.**
